# Impact of the Coronavirus Disease 2019 pandemic on neoadjuvant chemotherapy use in patients diagnosed with epithelial type ovarian cancer

**DOI:** 10.3389/fonc.2024.1290719

**Published:** 2024-03-27

**Authors:** Amrita Mukherjee, Natalie Shammas, Lanfang Xu, Kimberly L. Cannavale, Alec D. Gilfillan, Elizabeth A. Szamreta, Matthew Monberg, Melissa Hodeib, Chun R. Chao

**Affiliations:** ^1^ Department of Research and Evaluation, Kaiser Permanente Southern California, Pasadena, CA, United States; ^2^ Obstetrics and Gynecology, Adventist Health White Memorial Medical Center, Los Angeles, CA, United States; ^3^ Data Reporting and Analytics, MedHealth Statistical Consulting Inc., Solon, OH, United States; ^4^ Center for Observational and Real-World Evidence (CORE), Merck & Co., Inc., Rahway, NJ, United States; ^5^ Gynecology Oncology, Kaiser Permanente Southern California, Riverside, CA, United States

**Keywords:** COVID-19 pandemic, ovarian cancer, neoadjuvant chemotherapy, surgery, race/ethnicity

## Abstract

**Introduction:**

The Coronavirus Disease 2019 (COVID-19) pandemic posed critical challenges in providing care to ovarian cancer (OC) patients, including delays in OC diagnosis and treatment initiation. To accommodate for delays in OC surgery, the Society of Gynecologic Oncology (SGO) recommended preferential use of neoadjuvant chemotherapy during the pandemic. The purpose of this study was to assess the association of the COVID-19 pandemic with neoadjuvant chemotherapy use in patients diagnosed with OC.

**Methods:**

This retrospective cohort study included patients diagnosed with stage II-IV ovarian cancer of epithelial subtype between 01/01/2017-06/30/2021 at Kaiser Permanente Southern California (KPSC), a large integrated healthcare system in the United States. Ovarian cancer patients diagnosed between 2017-2020 were identified from KPSC’s Surveillance, Epidemiology, and End Results (SEER)-affiliated cancer registry. Patients diagnosed in 2021 were identified from the electronic medical records (EMR) using ICD-10 diagnosis codes, followed by medical chart review to validate diagnosis and extract information on histology and stage at diagnosis. March 4, 2020 was used as the cut-off to define pre-pandemic and pandemic periods. Patients diagnosed with COVID-19 between OC diagnosis and treatment completion were excluded. Data on neoadjuvant chemotherapy use were extracted from the cancer registry and EMR, supplemented by chart review. Modified Poisson regression was used to evaluate the association of the pandemic with neoadjuvant chemotherapy use.

**Results:**

Of 566 OC patients, 160 (28.3%) were diagnosed in the pandemic period. Patients diagnosed in the pandemic period were slightly younger (mean age 62.7 *vs* 64.9 years, p=0.07) and had a higher burden of Charlson comorbidities (p=0.05) than patients diagnosed in pre-pandemic period. No differences in time to treatment initiation were observed by pandemic periods. Neoadjuvant chemotherapy use was documented in 58.7% patients during the pandemic period compared to 47.3% in pre-pandemic period (p=0.01). After adjusting for covariates, patients diagnosed in the pandemic period were 29% more likely to receive neoadjuvant chemotherapy than patients diagnosed in pre-pandemic period [RR(95%CI): 1.29(1.12-1.49)].

**Discussions:**

Ovarian cancer patients diagnosed in the COVID-19 pandemic were more likely to receive neoadjuvant chemotherapy than patients diagnosed before the pandemic. Future research on patient outcomes and trends in the post-pandemic period are warranted.

## Introduction

The COVID-19 pandemic posed unprecedented challenges in the delivery of healthcare worldwide; care in cancer patients was particularly affected. There were delays in cancer diagnosis and treatment initiation; modifications in cancer treatment regimens were also recommended to reduce potential COVID-19 exposure and cancer treatment-related complications, as well as to prioritize overall COVID-19 response (1). Patients with gynecologic cancers were no exception. Discontinuation of cervical cancer screening services, reduction in emergency visits and urgent referral in patients with suspected cancer, delays in treatment initiation, and alterations/postponement of surgical procedures were reported in gynecologic cancer patients (2–4).

While primary cytoreductive surgery is the first line treatment choice for patients with ovarian cancer, neoadjuvant chemotherapy followed by interval cytoreductive surgery is not inferior to primary debulking surgery in advanced ovarian cancer patients (5, 6). During the COVID-19 pandemic, shortage of resources, including personal protective equipment (PPE) and ventilators, and repurposing of operating rooms as critical care units affected the standard of care (7). After weighing the risks and benefits of COVID-19 infection and surgical delay in gynecologic oncology patients, the American College of Surgeons (ACS) categorized gynecologic cancer cases, including ovarian cancer cytoreductive surgery, as ‘semi-urgent’ (8, 9). At the same time, to accommodate for delays in ovarian cancer surgery and to reduce harm, the Society of Gynecologic Oncology (SGO) recommended the use of neoadjuvant chemotherapy during the pandemic (8); SGO’s recommendation to use neoadjuvant chemotherapy preferentially was consistent with recommendations from other international gynecologic oncology societies (10, 11).

Studies conducted in Portugal and Netherlands have reported an increased administration of neoadjuvant chemotherapy in patients with advanced ovarian cancer during the COVID-19 pandemic, compared to pre-pandemic periods (2, 12). However, to our knowledge, evidence on the impact of the COVID-19 pandemic on neoadjuvant chemotherapy use in the United States (US) has not been reported. Our aim was to assess the association of the COVID-19 pandemic with neoadjuvant chemotherapy use in patients diagnosed with ovarian cancer at Kaiser Permanente Southern California (KPSC).

## Materials and methods

### Study design, setting, and subjects

This retrospective cohort study included patients diagnosed with incident epithelial ovarian cancer between January 1st, 2017, and June 30^th^, 2021 at KPSC, an integrated healthcare delivery system serving more than 4.7 million racially and ethnically and socioeconomically diverse members in Southern California. Patients diagnosed with epithelial type ovarian cancer between 2017-2020 were identified from KPSC’s Surveillance, Endpoints, & End Results (SEER)-affiliated cancer registry. Patients diagnosed in 2021 were initially identified from KPSC’s electronic medical records (EMR) using ICD-10 diagnosis codes and subsequently confirmed by chart review. Stage II-IV patients were included in the study if they were aged 18-89 years and were active members of KPSC health plan at the time of ovarian cancer diagnosis. Patients were excluded if they a) had any prior ovarian cancer diagnosis, b) had another cancer diagnosis within six months prior to their incident ovarian cancer diagnosis, c) had missing information on age at diagnosis, cancer stage, and/or race and ethnicity, d) had less than 12 months KPSC membership prior to ovarian cancer diagnosis (i.e., insufficient data to assess comorbidity burden), e) terminated KPSC membership within 12 months after ovarian cancer diagnosis, f) did not receive ovarian cancer treatment at KPSC, or g) were diagnosed with COVID-19 anytime between ovarian cancer diagnosis and ovarian cancer treatment completion (first course of treatment). The study was approved by KPSC’s institutional review board (IRB). Due to the use of secondary, de-identified data, the requirement for written or verbal consent was waived.

### Data collection

Data on ovarian cancer, including date of diagnosis, cancer stage, histology type, and treatment were extracted from KPSC’s SEER-registry for patients diagnosed in 2017-2020. For 2021, ICD-10 diagnosis codes (were used to identify ovarian cancer patients from KPSC’s EMR. Medical chart reviews were conducted to validate ovarian cancer diagnosis and to extract information on cancer histology and stage in patients identified from the EMR. Data on neoadjuvant chemotherapy use were extracted from the cancer registry and EMR, supplemented by chart review. Chart reviews were also conducted to understand the rationale behind neoadjuvant chemotherapy use in a small number of stage II ovarian cancer patients. Data on sociodemographic and other clinical variables were extracted from the EMR.

### Outcome, exposure, and covariates of interest

Use of neoadjuvant chemotherapy (yes/no) was the outcome of interest. Exposure of interest was the COVID-19 pandemic period. March 4, 2020 was used as the cut-off to define pre-pandemic and pandemic periods; this cut-off was based on date of implementation of stay-at-home order in California. Covariates of interest included age at cancer diagnosis, race and ethnicity, cancer stage, KPSC membership years prior to ovarian cancer diagnosis, Charlson Comorbidities Index, and Neighborhood deprivation index (NDI). Cancer stage included FIGO stages II-IV. Modified Charlson Comorbidities Index (unweighted) was calculated based on comorbidities recorded in the EMR within 12 months prior to ovarian cancer diagnosis (13); diagnosis of ovarian cancer was excluded from Charlson Comorbidities Index calculations. Neighborhood deprivation index (in quartiles), a measure for neighborhood socioeconomic status (SES), was geocoded based on patients’ home addresses and was based on the American Community Survey – Census Bureau data.

### Statistical analysis

Descriptive statistics comparing pre-pandemic and pandemic period were reported using frequency (percentage) or mean (standard deviation); Chi-square and t-test p-values were reported, as appropriate. Modified Poisson regression with robust variance was used to evaluate unadjusted and adjusted association of the pandemic with neoadjuvant chemotherapy use. Covariates with score test p-value <0.10 in the unadjusted models were included as potential confounders in the adjusted model. To check for effect modification by race and ethnicity, we evaluated the interaction between race and ethnicity and pandemic periods in the adjusted model. In sensitivity analysis, patients with stage II cancer, as well as patients with low-grade serous carcinoma were excluded. Level of significance was set at 0.05 and two-sided p-values were reported. All analyses were conducted in SAS version 9.4 (Cary, NC).

## Results

**Figure 1:**
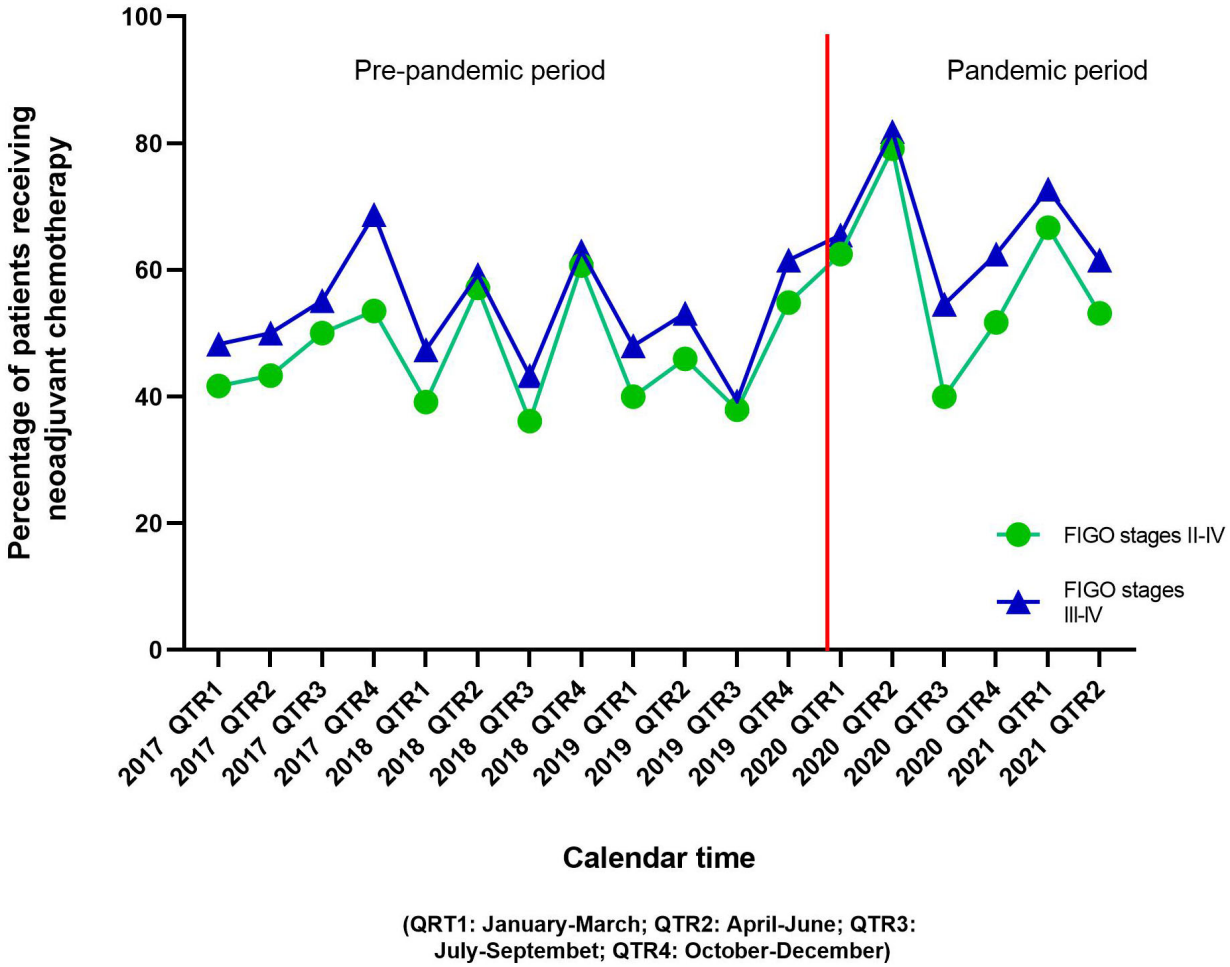
Distribution of ovarian cancer patients receiving neoadjuvant chemotherapy before and after the advent of the COVID-19 pandemic.

Sociodemographic and clinical characteristics of the study population are shown in **Table 1**. Of 566 stage II-IV ovarian cancer patients included, 406 (71.7%) were diagnosed during the pre-pandemic period and the rest [160 (28.3%)] were diagnosed during the pandemic period. Patients diagnosed during the pandemic period were slightly younger than patients diagnosed in the pre-pandemic period [mean age 62.7 *vs* 64.9 years, respectively, p-value=0.07]. Overall, 47.0% patients were non-Hispanic white, followed by 34.1% Hispanic patients, 10.8% Asian/Pacific Islander/other races, and 8.1% non-Hispanic black patients. No differences in race and ethnicity were observed by the pandemic periods. Overall, 85.5% patients were diagnosed at FIGO stage III-IV. Mean (std dev) time from diagnosis to neoadjuvant treatment initiation was 22.3 (24.5) days, with no differences observed by pandemic periods (p-value=0.17). More than one-third of the patients diagnosed during the pandemic period (38.8%) had more than two Charlson comorbidities compared to 30.5% patients diagnosed during the pre-pandemic period (p-value=0.05). Overall, 50.5% patients received neoadjuvant chemotherapy; 58.7% patients diagnosed in the pandemic period received neoadjuvant chemotherapy compared to 47.3% in the pre-pandemic period (p-value=0.01). Proportion of patients who received neoadjuvant chemotherapy by calendar period (quarters) is shown in **Figure 1**. The increase in neoadjuvant chemotherapy use corresponded with the first and second waves of the pandemic in California.

**Table 1:** Sociodemographic and clinical characteristics of patients diagnosed with stage II-IV ovarian cancer of epithelial origin at Kaiser Permanente Southern California by pandemic periods.

	Pre-pandemic period (N=406, 71.7%)	Pandemic period (N=160, 28.3%)	Total (N=566)	p-value
**Age at diagnosis (years) ** Mean (SD)	64.9 (11.38)	62.7 (12.15)	64.3 (11.64)	0.07
**Age at Diagnosis ** 18<=Age<55 55<=Age<65 65<=Age<75 75<=Age<89	78 (19.2%) 119 (29.3%) 119 (29.3%) 90 (22.2%)	45 (28.1%) 43 (26.9%) 45 (28.1%) 27 (16.9%)	123 (21.7%) 162 (28.6%) 164 (29.0%) 117 (20.7%)	0.11
**Race and Ethnicity ** Asian/Pacific Islander/Others Non-Hispanic black Hispanic Non-Hispanic white	41 (10.1%) 28 (6.9%) 139 (34.2%) 198 (48.8%)	20 (12.5%) 18 (11.3%) 54 (33.8%) 68 (42.5%)	61 (10.8%) 46 (8.1%) 193 (34.1%) 266 (47.0%)	0.23
**FIGO Stage ** Stage II Stage III Stage IV	57 (14.0%) 223 (54.9%) 126 (31.0%)	25 (15.6%) 84 (52.5%) 51 (31.9%)	82 (14.5%) 307 (54.2%) 177 (31.3%)	0.84
**Cancer Histology ** Clear cell carcinoma Endometrioid carcinoma Mixed epithelial Mucinous carcinoma Serous carcinoma Squamous carcinoma Transitional cell or Brenner carcinoma Undifferentiated or other epithelial	19 (4.7%) 17 (4.2%) 46 (11.3%) 7 (1.7%) 275 (67.7%) 1 (0.3%) 1 (0.3%) 40 (9.9%)	5 (3.1%) 12 (7.5%) 7 (4.4%) 3 (1.9%) 118 (73.8%) 0 (0.0%) 0 (0.0%) 15 (9.4%)	24 (4.2%) 29 (5.1%) 53 (9.4%) 10 (1.8%) 393 (69.4%) 1 (0.2%) 1 (0.2%) 55 (9.7%)	0.16
**Time treatment initiation (days)** Mean (SD)	23.3 (25.6)	19.8 (21.3)	22.3 (24.5)	0.17
**Prior KPSC Membership (years) ** 1to<5 5to<15 15to<30 30+	92 (22.7%) 119 (29.3%) 108 (26.6%) 87 (21.4%)	32 (20.0%) 52 (32.5%) 41 (25.6%) 35 (21.9%)	124 (21.9%) 171 (30.2%) 149 (26.3%) 122 (21.6%)	0.85
**Charlson Comorbidities Index** 0 1 2+	166 (40.9%) 116 (28.6%) 124 (30.5%)	67 (41.9%) 31 (19.4%) 62 (38.8%)	233 (41.2%) 147 (26.0%) 186 (32.9%)	0.05
**Neighborhood Deprivation Index (Quartiles) ** Highest SES Upper middle SES Lower middle SES Lowest SES	110 (27.1%) 79 (19.5%) 112 (27.6%) 105 (25.9%)	40 (25.0%) 37 (23.1%) 41 (25.6%) 42 (26.3%)	150 (26.5%) 116 (20.5%) 153 (27.0%) 147 (26.0%)	0.77
**Neoadjuvant chemotherapy use ** No Yes	214 (52.7%) 192 (47.3%)	66 (41.3%) 94 (58.7%)	280 (49.5%) 286 (50.5%)	0.01
**Neoadjuvant chemotherapy duration (days) ** Median (IQR)	64 (43.0, 106.0)	71 (43.0, 106.0)	64 (43.0, 106.0)	0.39
**Ovarian cancer treatment course ** Neoadjuvant chemo (no surgery) Neoadjuvant chemo + surgery Surgery only Surgery + adjuvant chemo	52 (12.8%) 140 (34.5%) 21 (5.2%) 193 (47.5%)	29 (18.1%) 65 (40.6%) 6 (3.8%) 60 (37.5%)	81 (14.3%) 205 (36.2%) 27 (4.8%) 253 (44.7%)	0.09

**Table 2** shows the unadjusted and adjusted association of the pandemic with neoadjuvant chemotherapy use in patients with Stage II-IV ovarian cancer. In the unadjusted model, patients diagnosed in the pandemic period were 24% more likely to receive neoadjuvant chemotherapy compared to patients in the pre-pandemic period [RR (95%CI): 1.24 (1.04-1.47)]. Compared to non-Hispanic white patients, Asian/Pacific Islander/other patients were less likely to receive neoadjuvant chemotherapy [RR (95%CI): 0.66 (0.47-0.94)]. Patients diagnosed at FIGO stage III and IV were more likely to receive neoadjuvant chemotherapy than patients diagnosed at FIGO stage II [RR (95%CI): 5.42 (2.64-11.12) and 9.07 (4.44-18.50), respectively]. Compared to patients with no Charlson comorbidities, patients with ≥2 Charlson comorbidities were 47% more likely to receive neoadjuvant chemotherapy [RR (95%CI): 1.47 (1.22-1.77)]. After adjusting for age, race and ethnicity, FIGO stage, and Charlson comorbidity index, patients in the pandemic period were 29% more likely to receive neoadjuvant chemotherapy compared to patients in the pre-pandemic period [RR (95%CI): 1.29 (1.12-1.49)].

**Table 2:** Unadjusted and adjusted association of the COVID-19 pandemic with neoadjuvant chemotherapy use in patients with stage II-IV ovarian cancer.

	Unadjusted Poisson model		Adjusted Poisson model*	
	[RR (95%CI)]	p-value**	[RR (95%CI)]	p-value**
**Pandemic periods ** Pre-pandemic Pandemic	Reference 1.24 (1.04-1.47)	0.01	Reference 1.29 (1.12-1.49)	<0.01
**Age at Diagnosis ** 18<=Age<55 55<=Age<65 65<=Age<75 75<=Age<89	Reference 1.52 (1.13-2.03) 1.79 (1.36-2.37) 1.67 (1.24-2.24)	<0.01	Reference 1.48 (1.14-1.92) 1.58 (1.22-2.05) 1.37 (1.03-1.81)	<0.01
**Race and Ethnicity ** Non-Hispanic white Asian/Pacific Islander/Others Non-Hispanic black Hispanic	Reference 0.66 (0.47-0.94) 1.00 (0.75-1.33) 0.89 (0.75-1.07)	0.06	Reference 0.72 (0.54-0.96) 0.87 (0.67-1.13) 0.97 (0.82-1.13)	0.09
**FIGO Stage ** Stage II Stage III Stage IV	Reference 5.42 (2.64-11.12) 9.07 (4.44-18.50)	<0.01	Reference 5.30 (2.60-10.81) 8.62 (4.23-17.53)	<0.01
**Prior KPSC Membership (years) ** 1to<5 5to<15 15to<30 30+	Reference 1.11 (0.87-1.42) 1.08 (0.84-1.40) 1.29 (1.01-1.65)	0.22	NA	NA
**Charlson Comorbidities Index ** 0 1 2+	Reference 1.14 (0.91-1.42) 1.47 (1.22-1.77)	<0.01	Reference 1.17 (0.96-1.42) 1.27 (1.06-1.52)	0.03
**Neighborhood Deprivation Index ** Highest SES Upper middle SES Lower middle SES Lowest SES	Reference 1.10 (0.87-1.39) 0.94 (0.74-1.19) 1.13 (0.91-1.41)	0.36	NA	NA

* Adjusted for age, race and ethnicity, FIGO stage, and Charlson comorbidities; [RR (95%CI)] = Risk Ratio (95% Confidence interval); ** Score test p-value; NA, not included in the adjusted model.

Of the covariates included in the adjusted model, age, cancer stage, and Charlson comorbidities were associated with neoadjuvant chemotherapy use. Older patients were more likely to receive neoadjuvant chemotherapy than patients aged <55 years [RR (95%CI): 1.48 (1.14-1.92) and 1.58 (1.22-2.05) for age groups 55to<65 and 65to<75 years, respectively]. Patients diagnosed at FIGO stages III and IV were more likely to receive neoadjuvant chemotherapy than stage II patients [RR (95%CI): 5.30 (2.60-10.81) and 8.62 (4.23-17.53), respectively]. Patients with ≥2 Charlson comorbidities were 27% more likely to receive neoadjuvant chemotherapy than patients with no Charlson comorbidities [RR (95%CI): 1.27 (1.06-1.52). Association of race and ethnicity with neoadjuvant chemotherapy was modified by pandemic periods; Hispanic and non-Hispanic white patients were 46% and 27% more likely to receive neoadjuvant chemotherapy during the pandemic period compared to the pre-pandemic period [RR (95%CI): 1.46 (1.13-1.88) and 1.27 (1.04-1.54), respectively] (data not shown).

Sensitivity analysis was limited to patients with advanced ovarian cancer (FIGO stages III-IV, n=484). After adjusting for age, race and ethnicity, FIGO stage, and Charlson Comorbidities Index, patients in the pandemic period were 28% more likely to receive neoadjuvant chemotherapy compared to patients in the pre-pandemic period [RR (95%CI): 1.28 (1.11-1.48)]. Patients with FIGO stage IV cancer were 63% more likely to use neoadjuvant chemotherapy than patients with stage III cancer [RR (95%CI): 1.63 (1.42-1.88)]. Older age and ≥2 Charlson comorbidities were also associated with higher neoadjuvant chemotherapy use (data not shown).

## Discussion

The COVID-19 pandemic was associated with increased use of neoadjuvant chemotherapy in patients diagnosed with advanced ovarian cancer in an integrated healthcare delivery system in the US. No statistically significant differences in cancer stage at diagnosis and time to treatment initiation were observed before and during the pandemic. To our knowledge, no previous studies have reported on the impact of the COVID-19 pandemic on neoadjuvant chemotherapy use in ovarian cancer patients in the US.

Studies conducted in Europe have reported an increase in neoadjuvant chemotherapy use in ovarian cancer patients after the onset of the pandemic (2, 12). Our findings are consistent; we observed a 29% increased use of neoadjuvant chemotherapy in patients diagnosed during the pandemic period compared to pre-pandemic patients. This finding has important clinical implications as it highlights the adoption of SGO’s modified treatment recommendations in ovarian cancer patients during the pandemic, in the US. However, unlike Algera’s and Antunes’ studies (2, 12), we reported the adjusted association of the COVID-19 pandemic with neoadjuvant chemotherapy. In the adjusted model, we observed that patients in the older age groups, patients diagnosed at FIGO stages III and IV, and patients with a higher comorbidity burden were more likely to receive neoadjuvant chemotherapy than their respective counterparts. Although neoadjuvant chemotherapy is mostly recommended in patients with advanced stage (stages III-IV) ovarian cancer, 8.5% of stage II patients in our cohort received neoadjuvant chemotherapy (data not shown); these patients were either too sick and frail to receive primary cytoreductive surgery, had a high comorbidity burden, or did not consent to surgery as the first-line treatment. When we limited our analysis to stage III-IV patients, more advanced cancer stage was still associated with increased neoadjuvant chemotherapy use. These findings are expected based on clinical management guidelines for ovarian cancer (14). Older age, advanced cancer stage, and comorbidities make these patients poor candidates for optimal cytoreductive surgery, and increase their vulnerability to COVID-19 infection and severity (10, 14, 15).

So far, evidence on COVID-19’s impact on ovarian cancer treatment patterns in the US is limited. Frey and colleagues reported treatment delay and change/cancellation of treatment regimens in gynecologic cancer patients receiving care at three New York city hospitals, although they did not assess trends in neoadjuvant chemotherapy use by pandemic periods (3). While Frey and colleagues observed delay in treatment initiation or change in treatment protocols in 38.7% of their study population due to the pandemic, they were unable to determine if treatment delays resulted from hospital policies or from patient-related factors (3). We did not observe any difference in time to treatment initiation before and after the onset of the pandemic. This might be attributed to KPSC’s integrated delivery of care model that ensures timely multidisciplinary care. It is also important to note that all patients included in our study were insured. Since we were interested in assessing the impact of the pandemic on neoadjuvant chemotherapy independent of patient’s COVID infection status, unlike Frey’s study, we only included patients who did not have any COVID-19 infection/diagnosis between cancer diagnosis and completion of first course of treatment.

Our study had some limitations. We did not have information on neoadjuvant chemotherapy dose; we were unable to assess if the COVID-19 pandemic impacted the recommended neoadjuvant chemotherapy dose in ovarian cancer patients. However, when we compared duration of neoadjuvant chemotherapy use before and during the pandemic, no statistically significant differences were observed. We did not have individual level SES data, but we used NDI as a proxy for SES. Our study population consisted of insured patients within an integrated health care system, hence, our findings may not be generalizable to other ovarian cancer cohorts. However, we included a racially and ethnically diverse cohort of ovarian cancer patients, and our extensive EMR database allowed us to capture the complete clinical characteristics of our patient population.

In conclusion, our study provided a snapshot of the impact of the pandemic on ovarian cancer care in the US, without the confounding effects of access to care. We observed increased neoadjuvant chemotherapy use during the pandemic compared to the pre-pandemic period in patients with advanced stage cancer. Future studies are needed to assess the impact of the pandemic on treatment patterns and cancer outcomes, including response to cancer treatments and survival in patients with ovarian cancers.

## Data availability statement

Data analyzed during the current study are not publicly available due to the nature of the database (Electronic health records). Requests to access these datasets should be directed to CC, Chun.R.Chao@kp.org.

## Ethics statement

The studies involving humans were approved by Kaiser Permanente Southern California Institutional Review Board. The studies were conducted in accordance with the local legislation and institutional requirements. The ethics committee/institutional review board waived the requirement of written informed consent for participation from the participants or the participants’ legal guardians/next of kin because secondary, de-identified data were used.

## Author contributions

AM: Investigation, Methodology, Validation, Writing – original draft. NS: Validation, Writing – review & editing. LX: Data curation, Formal analysis, Investigation, Methodology, Validation, Writing – review & editing. KC: Data curation, Investigation, Project administration, Validation, Writing – review & editing. AG: Data curation, Investigation, Validation, Writing – review & editing. ES: Conceptualization, Methodology, Writing – review & editing. MM: Conceptualization, Methodology, Writing – review & editing. MH: Investigation, Methodology, Validation, Writing – review & editing. CC: Conceptualization, Funding acquisition, Investigation, Methodology, Supervision, Writing – review & editing.
